# Comparison of soil microbial community structure and function for karst tiankeng with different degrees of degradation

**DOI:** 10.1002/ece3.9615

**Published:** 2022-12-08

**Authors:** Cong Jiang, Hui Zeng

**Affiliations:** ^1^ School of Urban Planning and Design Peking University Shenzhen Graduate School, Peking University Shenzhen China

**Keywords:** biodiversity, functional prediction, habitat changes, karst, random molecular ecological network

## Abstract

Karst tiankengs are oases in degraded karst landscapes and act as repositories for biodiversity conservation; however, knowledge about the bacterial and fungal structure and function of the karst tiankeng ecosystems is limited. This study investigated the microbial communities in three different tiankeng (nondegraded, moderately degraded, and heavily degraded tiankeng) by Illumina NovaSeq sequencing. We found that the degradation of karst tiankeng can lead to changes in microbial community structure and functions, while there are differences in bacterial and fungal responses. There were significant differences in bacterial and fungal community composition and beta diversity in the three tiankeng soils. Random molecular ecological network analysis results indicated that a more complex and stable bacterial network existed in nondegraded tiankeng, while more complex fungal networks existed in moderately degraded tiankeng. The keystones of *Proteobacteria*, *Actinobacteria*, *Acidobacteria*, *Ascomycota*, and *Basidiomycota* played essential roles in maintaining soil function and stability. The functional profiles revealed that tiankeng habitat changes may affect microbial survival strategies, such as increasing gene abundance associated with the carbon cycle. To our knowledge, this is the first report on bacterial and fungal communities in different degrees of karst tiankeng, which provides crucial insights into our understanding of the microbial communities' structure and potential function in karst tiankeng ecosystems.

## Graphical abstract

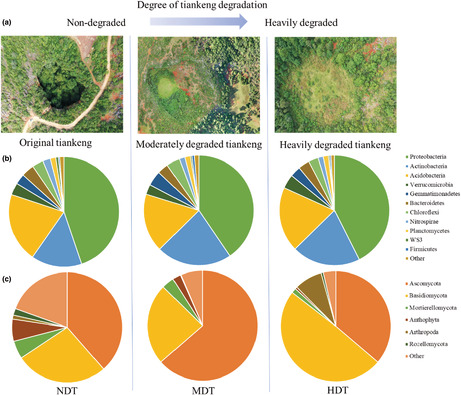

## 1 INTRODUCTION

The global karst area is about 22 million km^2^, accounting for 12% of the world's land area (Jiang et al., 2014). Due to the fragile geotechnical system and binary hydrological structure, the karst ecosystem is of global importance, supporting a distinct ecosystem. Soil erosion, poor soils, and biodiversity loss are typical features of karst ecosystems (Clements et al., 2006). Moreover, shallow soil layers of karst are rich in organic matter, which makes karst soil C likely to be more sensitive to global climate change (Ahmed et al., 2012). However, karst ecosystems are not always barren, and karst tiankeng are “oases” in the karst ecosystem.

Karst tiankeng are a type of large negative terrain with huge volumes and deep into the surface, and was first discovered in the karst landscape of southern China in the 1990s (Zhu & Waltham, 2005a, 2005b). Constrained by the trapped terrain, the internal habitat of karst tiankeng is independent of the external environment (Shui et al., 2015). The unique habitats within the tiankeng can meet the needs of more species with different ecological niches, and preserve unique flora, fauna, and microbial resources (Jiang et al., 2022; Pu et al., 2021; Su et al., 2017). Karst tiankeng is a conservation reservoir of biodiversity and sanctuary for endangered species in the local region. According to the morphology of karst tiankeng, it can be divided into nondegraded tiankeng (original tiankeng) and degraded tiankeng (Chen et al., 2009). Degraded tiankeng is characterized by the destruction of the integrity of the surrounding vertical rock walls. The destruction of the vertical cliffs means that the internal environment of the karst tiankeng changes (e.g., hydrothermal, temperature, light), exposing these systems to increased disturbance events.

Soil microorganisms are the engines in the biogeochemical cycles and are essential for the decomposition of organic matter and nutrient cycling processes in soils (Balser & Firestone, 2005; Fierer, 2017). In the karst area, soil microorganisms play an important role in the carbon and nitrogen cycles and mediate nutrient transfer between plant and soil (Wang et al., 2020; Xue et al., 2020). Interactions between aboveground and subsurface communities drive ecosystem diversity and determine the succession and development of biomes (van der Putten et al., 2013). Plants, soils, and microorganisms interact closely, and habitat changes must be accompanied by changes in soil microbial communities. The degradation of karst tiankeng is caused by the secondary collapse of the vertical cliff wall due to the instability of the carbonate rock, and the surrounding cliff wall is gradually buried by the accumulation (Zhu & Waltham, 2005a, 2005b). Karst tiankeng degradation is accompanied by the destruction of unique habitats within the tiankeng, which may eventually converge with the degraded karst landscape on the surface. Changes in habitats within tiankeng inevitably lead to changes in the structure and function of microbial communities and also reflect changes in karst tiankeng ecosystems. Therefore, it is of great significance to study the differences in microbial communities between tiankeng with different degrees of degradation for the conservation and ecological evaluation of karst tiankeng.

Thus, we studied the soil bacterial and fungal communities at three karst tiankeng (nondegraded, moderately degraded, and heavily degraded tiankeng). The main purposes of this study are (i) to reveal the effects of karst tiankeng degradation on soil microbial community (bacterial and fungal) structure and function; (ii) to evaluate the impact of karst tiankeng degradation on microbial interaction patterns and network stability.

## 2 MATERIALS AND METHODS

### 2.1 Study area and soil sampling

We conducted this study in 2021 in Zhanyi district, Qujing City, Yunnan Province, China (25°35′–25°57′N, 103°29′–103°39′E). The study was performed at Haifeng natural reserve (Figure S1). The study area has dozens of karst tiankengs. The average annual rainfall and temperature were 1081.6 mm and 14°C, respectively. Most rainfall was concentrated in summer and autumn. The soil in the region is Yunnan red soil.

**FIGURE 1:**
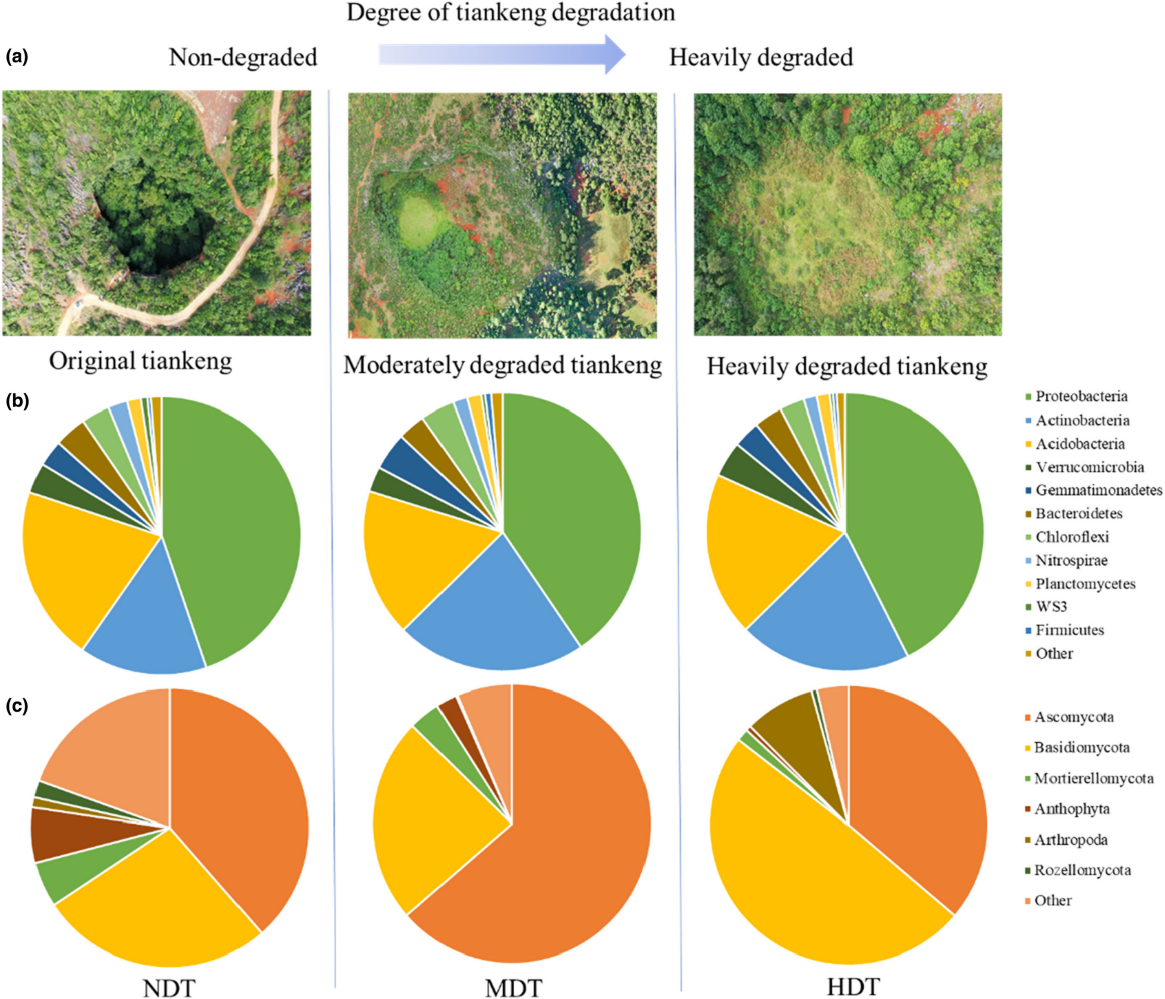
Aerial photographs of the study sites of karst tiankeng with different degrees of degradation (a); The microbial community composition of major taxa (at the phylum level) of bacteria (b) and fungi (c) of karst tiankeng with different degrees of degradation.

Based on a method described by Chen et al. (2009), we selected three karst tiankeng, including nondegraded tiankeng (NDT), moderately degraded tiankeng (MDT), and heavily degraded tiankeng (HDT; Figure 1a). The bottoms of moderately degraded tiankeng and heavily degraded tiankeng were once farmed and have been restored naturally for more than 30 years. Each tiankeng degradation level was set for eight replicates (10 m × 10 m^2^) of three randomly established quadrats (1 m × 1 m^2^). The vegetation species and number within the sampling sites (10 m × 10 m^2^) were identified and recorded. The Shannon–Wiener (*H*) and Margalef richness (*D*) indexes were calculated, and the specific calculation methods have been described in detail elsewhere (Peng et al., 2019). The soil samples of 0–15 cm depth were collected in a five‐point pattern and mixed as a composite sample. The collected soil sample was homogenized, sieved (2 mm), and divided into two equal subsamples. One subsample was stored at −80°C for DNA extraction, and the other subsample was for soil physicochemical analyses. The soil physicochemical properties, including soil bulk density (BD), soil water content (SWC), soil organic carbon (SOC), total nitrogen (TN), available nitrogen (AN), total phosphorus (TP), available phosphorus (AP), and pH were determined as described in Bao (2000).

### 2.2 DNA extraction and sequencing

Total DNA was extracted using the CTAB method (Niemi et al., 2001). The DNA purity was examined with 1% agarose gels, and clear bands indicate the high purity of the extracted DNA. The DNA concentration was examined with NanoDrop 2000 Spectrophotometers (Thermo Scientific). The V4 region of the bacterial gene and the ITS2 region of the fungal rRNA gene were amplified on Phusion® High‐Fidelity PCR Master Mix (New England Biolabs) with the primers 515F (5′‐GTGCCAGCMGCCGCGGTAA‐3′) and 806R (5′‐GGACTACHVGGGTWTCTAAT‐3′) for 16S rRNA (Zhang, Ding, et al., 2018) and ITS3F (5′‐GCATCGATGAAGAACGCAGC) and ITS4R (5′‐TCCTCCGCTTATTGATATGC) for ITS (Jamil et al., 2020). The sequencing libraries were generated using TruSeq® DNA PCR‐Free Sample Preparation Kit (Illumina). Raw sequence reads were obtained from Illumina NovaSeq 6000 PE250 platform (Illumina), and deposited in the NCBI SRA with the accession number PRJNA891650 for 16S sequences and PRJNA891697 for ITS sequences.

The raw data FASTQ files were filtered and analyzed by the QIIME2 system (Vázquez‐Baeza et al., 2013). The chimeric sequences were identified by QIIME2 DADA2 plugin after quality filtered, trimmed, de‐noised, and merged. The bacterial and fungal representative sequences were taxonomically classified by aligned against the GREENGENES 13_8 and UNITE database, respectively (Bokulich et al., 2018).

### 2.3 Bioinformatics analysis

The Chao1 richness and Shannon diversity index were calculated using the core‐diversity plugin within QIIME2 (http://qiime.org/index.html). Bray–Curtis (BC) distance matrix between the microbial communities taxonomic or functional across study sites was visualized via principal coordinate analysis (PCoA; Gabarrón‐Galeote et al., 2013; Zhou et al., 2019). The microbial community dissimilarity was examined by analysis of similarities (ANOSIM). Comparisons of soil physicochemical, vegetation diversity, and microbial diversity index between the different degrees of degradation were examined by ANOVA analyses. The redundancy analysis (RDA) was used to elucidate the association of microbial communities and soil factors using the R (v 4.1.2) package “vegan” (Dixon, 2003). The microbial community changes between the different tiankeng were examined by nonlinear regression in R (v 4.1.2). The metabolic functional profiles of karst tiankeng microbial communities were predicted with PICRUSt (Langille et al., 2013), focusing on C and N cycle genes. The KEGG orthologues level 2 data were downloaded for the main functional pathways analysis.

The construction of the molecular ecological networks (MEN) for karst tiankeng microbial communities was based on the principle of the Molecular Ecological Network Analyses Pipeline (Zhou et al., 2010). Identifying the appropriate similarity threshold (St) and construction of molecular ecological networks was based on the random matrix theory (RMT; Deng et al., 2012). The network was visualized by Cytoscape (v 3.9.0). The topological roles of nodes in the network were represented by within‐module connectivity (Zi) and among‐module connectivity (Pi; Deng et al., 2012). The connectors (Zi < 2.5 and Pi ≥ 0.62), module hubs (Zi ≥ 2.5 and Pi ≤ 0.62), and network hubs (Zi ≥ 2.5 and Pi ≥ 0.62) were considered generalists, which acted as keystone taxa in the network of the microbial community (He et al., 2017).

## 3 RESULTS

### 3.1 Soil physicochemical and vegetation characteristics of karst tiankeng with different degrees of degradation

The soil physicochemical properties showed significant differences among the degraded tiankeng (Table 1). The BD value of MDT was significantly higher than that in NDT and HDT (*p* < .05). Compared with MDT, the content of TN was significantly higher in NDT (*p* < .05). Compared with HDT, the contents of TP and AP were significantly higher in NDT (*p* < .05). The NDT soils had higher SWC and no significant differences between MDT and HDT soils. The soil pH had a narrow variation, with a range of 6.18–6.79. The vegetation characteristics changed significantly as well (Table S1). The highest vegetation Shannon–Wiener and Margalef richness were observed in MDT (*p* < .05).

**TABLE 1:** The soil physicochemical properties of nondegraded tiankeng (NDT), moderately degraded tiankeng (MDT), and heavily degraded tiankeng (HDT).

	BD (g cm^3^)	SWC (%)	SOC (g kg^−1^)	TN (g kg^−1^)	TP (mg kg^−1^)	AP (mg kg^−1^)	AN (mg g^−1^)	pH
NDT	0.75 ± 0.22b	49.27 ± 8.62a	58.14 ± 20.45	4.80 ± 1.66a	848.38 ± 371.71a	1.09 ± 0.05a	347.21 ± 56.52a	6.79 ± 0.43a
MDT	1.07 ± 0.27a	34.22 ± 5.00b	53.25 ± 25.75	3.00 ± 1.96b	822.89 ± 313.58ab	1.06 ± 0.0.04ab	193.31 ± 109.78b	6.19 ± 0.24b
HDT	0.72 ± 0.10b	28.01 ± 8.33b	56.14 ± 14.27	3.88 ± 1.00ab	522.54 ± 155.78b	1.04 ± 0.03b	306.32 ± 91.28a	6.18 ± 0.44b

*Note*: Different lowercase letters shows statistically significant difference (*p* < .05).

Abbreviations: AN, Available nitrogen; AP, Available phosphorus; BD, Soil bulk density; HDT, Heavily degraded tiankeng; MDT, Moderately degraded tiankeng; NDT, Nondegraded tiankeng; SOC, Soil organic carbon; SWC, Soil water content; TN, Total nitrogen; TP, Total phosphorus.

### 3.2 Overall pattern of microbial community in karst tiankeng soils

In total, 13,304 bacterial ASVs and 9772 fungal ASVs were obtained from 24 soil samples. The highest bacterial and fungal ASVs numbers were observed in MDT and NDT, respectively. Among the bacterial ASVs, 3075, 3876, and 2918 specific ASVs were observed in the NDT, MDT, and HDT, respectively. A total of 3404, 3113, and 2051 specific fungal ASVs were observed in the NDT, MDT, and HDT, respectively (Figure S2).

**FIGURE 2:**
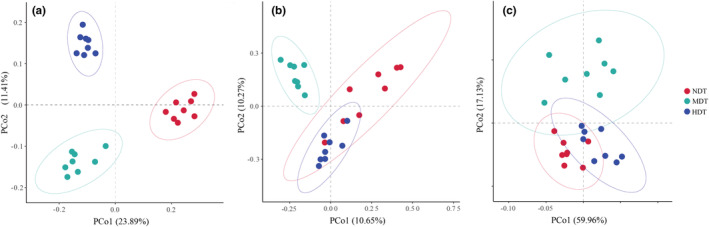
The principal coordinate analysis (PCoA) for the bacterial (a), fungal (b) communities, and functional gene (c) of karst tiankeng with different degrees of degradation.

The Alpha diversity (Chao1 richness index and Shannon–Wiener index) of the bacteria did not show significant differences among the degraded tiankeng. The highest fungi Chao1 richness was observed in NDT and the lowest values were observed in HDT (Figure S3; *p* < .05). At the phylum level, the bacterial communities were predominantly composed of the *Proteobacteria* (ranging from 40.48% to 44.79%), followed by *Actinobacteria* (ranging from 14.92% to 22.10%) and *Acidobacteria* (ranging from 17.19% to 20.38%). The *Ascomycota* (ranging from 36.22% to 63.61%) and *Basidiomycota* (ranging from 23.76% to 49.27%) were the most abundant fungi taxa (Figure 1). At the class level, the top 20 shared classes were screened (Figure S4). The most abundant bacteria were *Alphaproteobacteria* and *Betaproteobacteria*, which accounted for 17.98%–23.09% and 8.99%–11.34% in the different degraded tiankeng, respectively. The most abundant fungi were *Agaricomycetes*, which accounted for 12.03%–44.96%. The relative abundance of unclassified taxa was highest in NDT. The PCoA results showed that the bacterial communities of different degraded tiankeng were grouped separately (Figure 2a). The fungal communities in MDT and HDT clustered closely and separately from those in NDT (Figure 2b). The ANOSIM also showed that the bacterial communities (*r* = .471, *p* = .001) and fungal communities (*r* = .609, *p* = .001) differed significantly from the different degraded tiankeng (Figure S5).

Redundancy analysis (RDA) was used to reveal the effect of soil characteristics on the microbial community. The soil and vegetation characteristics can explain 53.76% and 53.73% of the variation in the bacterial and fungal communities, respectively (Figure S6). The bacterial communities were significantly related to BD, SWC, pH, and TN. The fungal communities were significantly related to BD, plant Shannon–Wiener, SWC, pH, and TN. The Mantel test results also indicated that soil characteristics were significantly related to changes in the microbial community (Table S2).

### 3.3 Microbial networks and keystone taxa of karst tiankeng soils with different degrees of degradation

To understand the potential microbe–microbe interactions in karst tiankeng, microbial random molecular ecological networks (MENs) were constructed. Both bacterial and fungal networks were scale‐free networks (*R* ^2^ values from .809 to .867). Both bacterial and fungal networks of NDT, MDT, and HDT exhibited a good modular structure with modularity indexes over 0.40. The highest values of average connectivity (avgK) (5.565) and lowest average geodesic distance (GD) (6.691) were observed in NDT, which indicated that the bacterial network of the NDT was more complex. Nevertheless, the fungal network was more complex in MDT (Table 2).

**TABLE 2:** Topological properties of bacterial and fungal networks of karst tiankeng soils with different degrees of degradation

	St	Nodes	Links	*R* ^2^ of power‐law	avgCC	GD	HD	avgK	Density	Modularity
Bacteria										
NDT	0.900	810	2254	.830	0.179	6.691	5.429	5.565	0.007	0.790
MDT	0.900	802	1731	.846	0.204	8.342	6.730	4.317	0.005	0.867
HDT	0.900	833	1596	.841	0.168	9.374	7.425	3.832	0.005	0.907
Fungi										
NDT	0.860	246	586	.867	0.186	4.653	3.755	4.764	0.019	0.638
MDT	0.860	209	693	.815	0.299	4.314	3.364	6.632	0.032	0.599
HDT	0.860	221	354	.809	0.176	6.032	4.740	3.204	0.015	0.799

Abbreviations: avgCC, Average clustering coefficient; avgK, Average degree; GD, Average path distance; HD, Harmonic geodesic distance; HDT, Heavily degraded tiankeng; MDT, Moderately degraded tiankeng; NDT, Nondegraded tiankeng.

**FIGURE 3:**
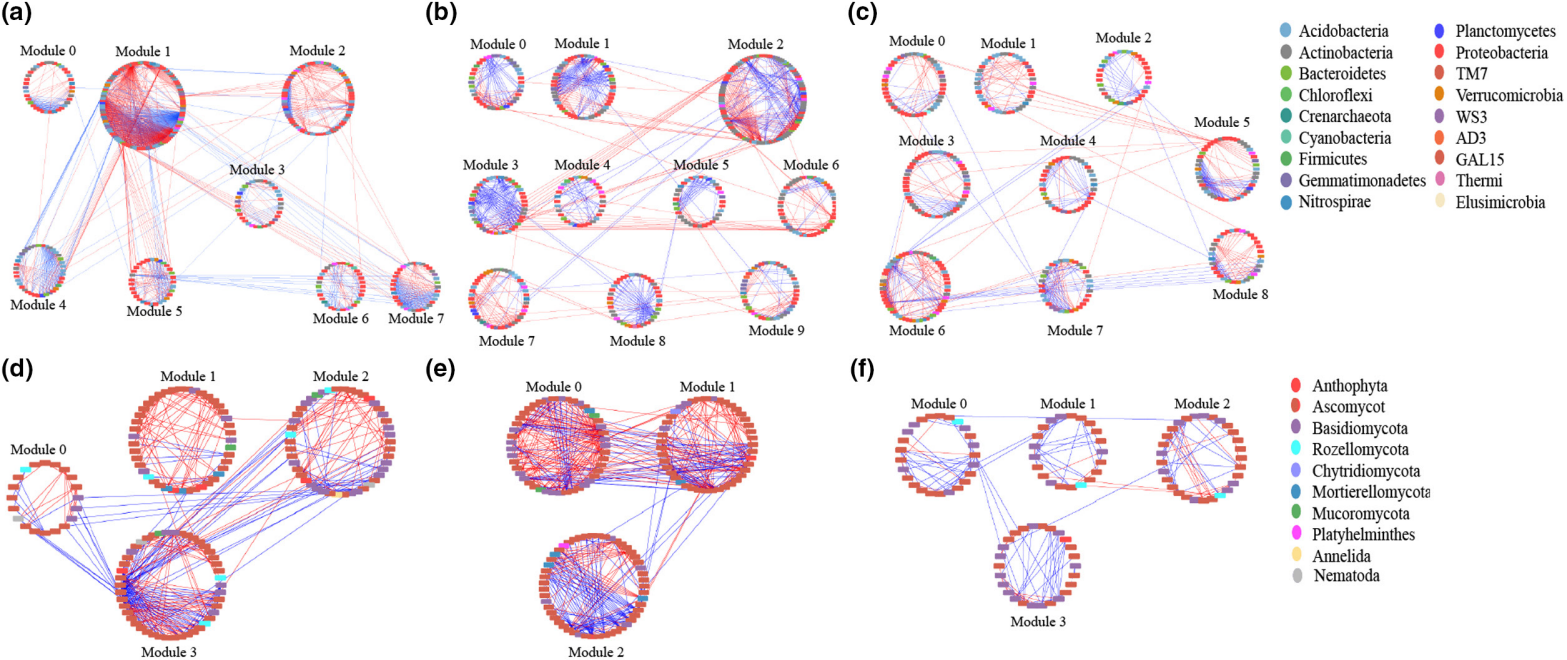
The highly connected modular of the random molecular ecological networks of bacteria (a–c) and fungi (d–f) of karst tiankeng with different degrees of degradation (a and d for NDT; b and e for MDT; c and f for HDT). Node color indicates different phyla. The red edges indicate positive interactions, and the blue edges indicate negative interactions.

In the bacterial network, 61 modules in NDT, 59 modules in MDT, and 64 modules in HDT were generated, and nodes number higher than 30 were identified as major modules (Figure 3). The max module sizes of the NDT (module 1) and MDT (module 2) achieved 114 and 103 nodes, respectively. In the NDT network, *Proteobacteria* dominated in all modules. In the MDT network, 10 modules were observed. *Proteobacteria* was dominated in most modules, except *Actinobacteria* was dominated in module 2. The module sizes of the HDT network were relatively homogeneous (ranging from 32 to 57), and nine modules were observed. In the fungal network, only 4, 3, and 4 major modules (nodes number > 20) were observed in NDT, MDT, and HDT, respectively. The max module sizes of the NDT (module 3) and MDT (module 2) were observed 46 and 51 nodes, respectively. For fungal network, *Ascomycota* was predominant in major modules of NDT and MDT. In the HDT network, module 2 was the max module in the HDT network, which was dominated by *Ascomycota* and *Basidiomycota*.

**FIGURE 4:**
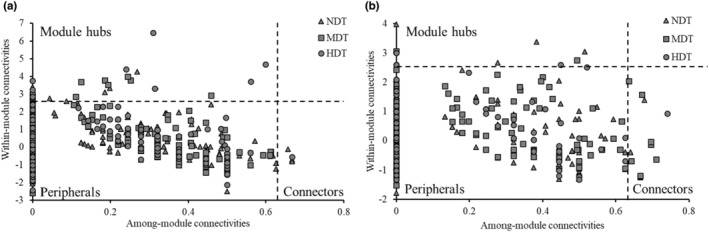
Identify keystone species within the networks according to their topological roles of bacteria (a) and fungi (b) of karst tiankeng with different degrees of degradation. Nodes with Zi ≥ 2.5 and Pi ≤ 0.62 was considered module hubs; nodes with Zi < 2.5 and Pi ≥ 0.62 was considered connectors.

The topological roles and functions of putative keystone taxa within the bacterial and fungal network were further evaluated (Figure 4; Tables S3 and S4). Among the nodes, no nodes were divided into the network hubs. In the NDT network, 22 keystone bacterial taxa (including 16 module hubs and six connectors) and nine keystone fungal taxa (including four module hubs and five connectors) were observed. Contrastingly, 23 keystone taxa (11 keystones bacterial and 12 keystones fungal taxa) and 19 keystone taxa (11 keystones bacterial and eight keystones fungal taxa) were observed in MDT and HDT, respectively. In the bacterial network, *Proteobacteria* and *Acidobacteria* worked as prominent phyla in all keystone taxa, which accounted for 72.73% of all keystone taxa. In addition, the other dominant phylum were *Actinobacteria*, *Chloroflexi*, *Bacteroidete*, and *WS3*. The bacterial genera were different among the different types of karst tiankeng, such as *Pseudonocardia*, *Pedomicrobium*, and *Candidatus_Solibacter* in NDT, *Hydrogenophaga*, *Mesorhizobium*, and *Rhodoplanes* in MDT, *Phenylobacterium*, and *Pilimelia* in HDT. In the fungal network, *Ascomycota* and *Basidiomycota* were predominant in all keystone taxa. *Agaricomycetes* class (*Russula_virescens* species and *Clavulinopsis*_sp) and *Sordariomycetes* class (*Mariannaea* genus) were the keystone taxa in the NDT network. *Eudicotyledonae* class (*Justicia* genus and *Knufia*_sp species) and *Dothideomycetes* class (*Sporormiella_mi* species and *Cladosporium* genus) in the MDT network. *Agaricomycetes* class (*Sebacina*_sp and *Agaricus_xanthodermus* species) was the keystone taxa in the HDT network.

### 3.4 The microbial potential functions of karst tiankeng with different degrees of degradation

**FIGURE 5:**
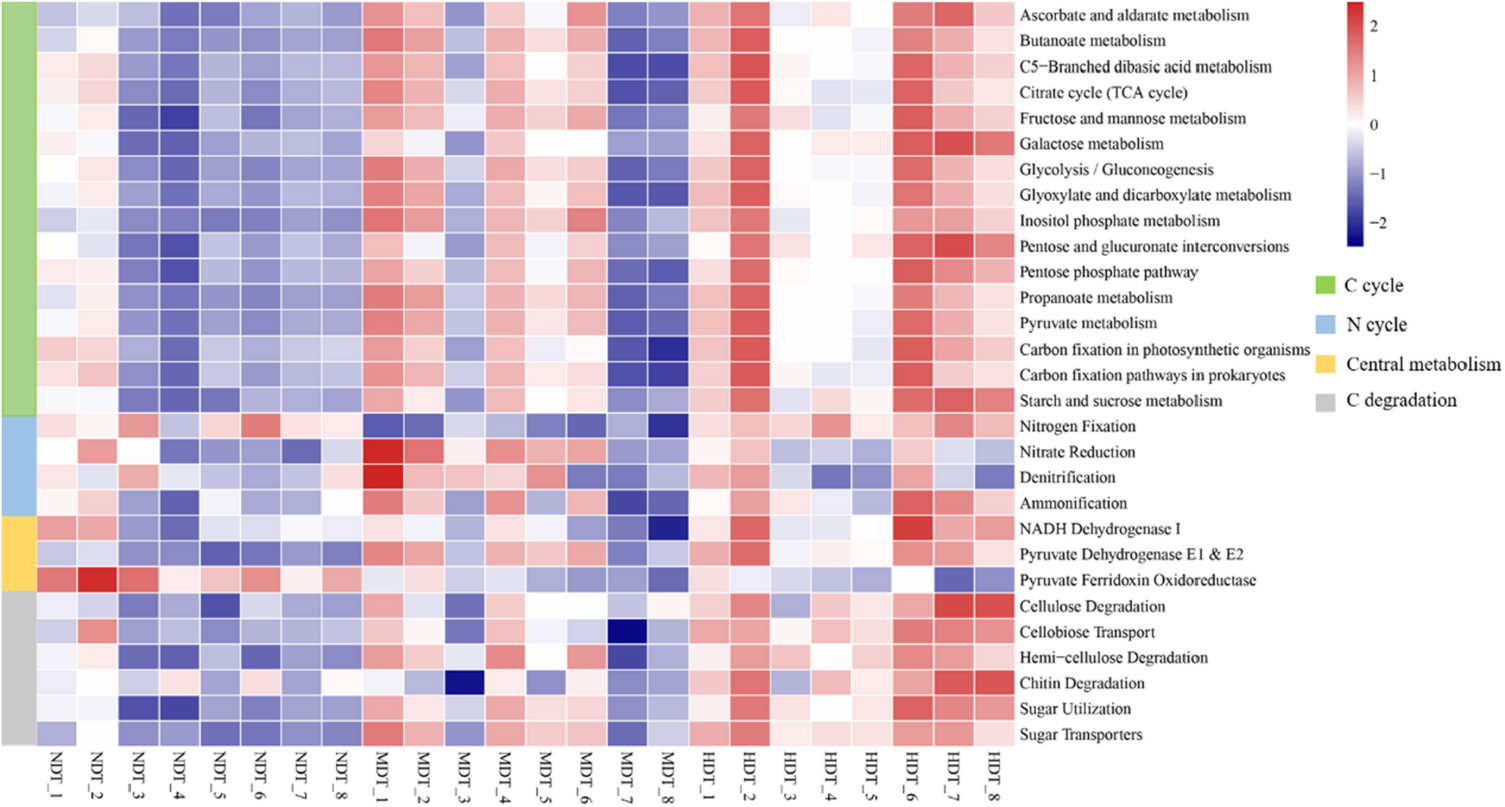
Heatmap of the functional genes (C and N cycles) for microbial communities from karst tiankeng with different degrees of degradation. Red represents a high abundance of functional genes and blue represents a low abundance of functional genes.

Based on the KEGG database, a total of 7461 KO genes were assigned in all soil metagenomes. Microbial potential functional changes among the three tiankeng were investigated via PCoA (Figure 2c), which showed partial differentiation in functional genes. At KEGG orthologue levels 2, a total of 23 functional pathways were observed (relative abundance >1%), and 20 functional pathways exhibited significant differences among the different types of karst tiankeng soil samples (Table S5). Among the three karst tiankeng, the energy metabolism and nucleotide metabolism were significantly higher in NDT (*p* < .05), metabolism of terpenoids and polyketides, amino acid metabolism and carbohydrate metabolism were significantly higher in MDT (*p* < .05). In addition, we found genes related to the C cycle (C cycle and C degradation) exhibited the lowest abundances in NDT and increased in abundance as tiankeng degradation increased (Figure 5; Figure S7). The genes related to nitrate reduction and denitrification showed higher abundance in MDT.

## 4 DISCUSSION

### 4.1 Differential microbial community taxonomic and functional composition of karst tiankeng with different degrees of degradation

In this study, we applied sequencing of 16S and ITS rRNA genes and soil metagenomics to explore the taxonomic and functional information of karst tiankeng with different degrees of degradation. The results showed that karst tiankeng degradation significantly alters soil microbial community taxonomic and function. In relatively isolated karst tiankeng ecosystems, the adaptability of microbial species to specific habitats was reflected by soil microbial community taxonomic and functional composition. Although our results showed no significant difference in bacterial community diversity among the three karst tiankengs, the Chao1 richness of fungal community decreased with tiankeng degradation and the lowest values were observed in HDT (Figure S3). Abundant vegetation and fertile soils are often accompanied by high microbial community diversity (Guo et al., 2018). Our previous research has demonstrated that microbial community diversity within the tiankeng was significantly higher than outside the tiankeng (Jiang et al., 2022). Due to the lack of vertical rock wall isolation, the habitat inside the tiankeng tends to be consistent with the habitat outside the tiankeng (typical karst degraded landscape), which may affect microbial community diversity and richness. Both the composition of bacterial and fungal communities were different among the three tiankeng types (Figure 1a, Figure S4). The phyla of *Proteobacteria* and *Actinobacteria* (bacteria) and *Ascomycota* and *Basidiomycota* (fungi) were dominated in three karst tiankeng. This finding is consistent with previous studies conducted in the karst area (Pu et al., 2019; Xiao et al., 2022). However, the same microbial taxa exhibit differences in abundance with different tiankeng types. Soil microbes respond to habitat changes by changing the abundance of specific populations (Jangid et al., 2011). For example, *Proteobacteria* and *Bacteroidetes* were copiotrophic and prefer nutrient‐rich soil (Teixeira et al., 2010). The relative abundance of *Proteobacteria* and *Bacteroidetes* was higher in NDT, which might indicate that nondegraded tiankeng soil was more nutritious (Table 1). *Actinobacteria* were considered an indicator of drought‐tolerant (de Vries et al., 2018) and were higher in MDT and HDT, which might be due to the lower soil water content in MDT and HDT. *Gemmatimonadetes*, *Chloroflexi*, and *Firmicutes* usually live in disturbed and nutrient‐limited environments (Barnard et al., 2013), which have higher relative abundance in degraded tiankeng. The abundant bacterial phyla were controlled by different soil physicochemical factors. Typically, soil pH was the major driver of bacterial community and has been demonstrated in many ecosystems (Banning et al., 2011; Shen et al., 2013; Tripathi et al., 2018). pH is a strong factor that affects bacterial communities in this study (Figure S6a). Xue et al. (2017) suggested that a strong correlation between pH and soil bacterial community structure in karst environments may be due to the poor growth environment and relatively narrow growth tolerance exhibited by most bacterial taxa. In addition, the bacterial communities are also related to SWC, TN, and BD. Soil water content influences oxygen content and nutrient availability and consequently the soil microbial community (Banerjee et al., 2016). Soil bulk density can affect soil porosity and oxygen, which can also affect the microbial community (Cong et al., 2015). Saprophytic fungi consist mainly of the *Ascomycota* and *Basidiomycota* and are regarded to play a crucial role in soil organic matter decomposition processes. This result indicated that fungal communities that perform the decomposition and nutrient cycling might survive well in karst tiankeng. Previous studies reported that fungi can effectively utilize the soil C from plant litter and build a close relationship with plants (Dini‐Andreote et al., 2016), which is consistent with our results (Figure S6).

Changes in microbial community composition often lead to changes in community function (Le Roux, 2009). Microbial community predicted functional differentiation between different types of tiankeng (Figures 2c and 5; Table S5). Microbes in different habitats may change their survival strategies to adapt to the environment. Based on the KEGG database, we investigate genes associated with the C cycle, N cycle, C degradation, and central metabolism. Among the three karst tiankeng, the abundance of C cycle, C degradation, and central metabolism (NADH Dehydrogenase I and Pyruvate Dehydrogenase E1 & E2) genes were higher in HDT. Furthermore, the highest potential for nitrate reduction and denitrification was observed in MDT. The above results have an interesting explanation “nutritional restriction theory.” The theory suggests that the availability of resources is a key driver affecting microbial communities (Cherif & Loreau, 2007). When nutrients are limiting factors for microbial survival, the microbial community increases the abundance of C and N cycle genes to promote litter breakdown and nutrient cycling. However, when soil nutrients are relatively abundant, the microbial community reduces the abundance of these genes and eventually reaches a stable state. The microbial communities of karst degraded tiankeng are more susceptible to the influence of the external environment (e.g., nutrient limitation). The interior of the nondegraded tiankeng has a stable forest community (main hardwoods). Previous studies have shown that in the latter stages of vegetation community succession, microbes easily obtained nutrients, resulting in a lower abundance of C cycle and C decomposition genes (Zhong et al., 2018). Our study also suggested that the soil‐vegetation‐microbial system may arrive at a stable state within the nondegraded tiankeng.

### 4.2 The change of microbial co‐occurrence networks in response to karst tiankeng degradation

In relatively independent karst tiankeng, the interaction between microorganisms was driven by the survival of each species, which plays an important role in community stability. In this study, bacterial and fungal networks were robust, which suggested that the constructed networks were suitable for the interaction modes of the karst tiankeng microbial communities. In karst tiankeng soil, the bacterial network was more complex in nondegraded tiankeng, while the fungal network was more complex in moderately degraded tiankeng. This result indicated that bacteria and fungi respond differently to habitat changes. Numerous previous studies have revealed differential responses of bacteria and fungi to environmental change (Li et al., 2020; Shi et al., 2016; Zhang, Zhang, et al., 2018). Differences in the complexity of bacterial and fungal networks may be related to niche differentiation due to habitat changes in karst tiankeng. The environmental conditions in nondegraded tiankeng were more stable than in degraded tiankeng. Less environmental variability means weaker niche differentiation, and also means stronger microbial interactions (Faust & Raes, 2012; Ma et al., 2016). With the degradation of karst tiankeng, the environment inside and outside the tiankeng lacks barriers. The unique climate inside the tiankeng was destroyed, and also more susceptible to human disturbances. In particular, the heavily degraded tiankeng, and the environment inside and outside the tiankeng tends to homogenize. Soil degradation and human disturbances have a negative effect on the network structure of microbial communities (Tang et al., 2019; Xue et al., 2020). The complexity of microbial networks is often accompanied by greater community stability (Mougi & Kondoh, 2012). The simple network structure in the degraded tiankeng might suggest unstable and vulnerable soil microbial communities under climate change. The fungal network was more complex in moderately degraded tiankeng may be due to being tightly associated with vegetation communities (Adamczyk et al., 2019; Yang et al., 2017). In general, fungal diversity was positively correlated with vegetation diversity (Hiiesalu et al., 2017). The fungal communities and vegetation diversity were both higher in moderately degraded tiankeng (Figure S3B; Table S1). Higher species diversity may support the complexity of the fungal network.

Keystones play an important role in maintaining community stability with a high degree of diversity and complexity (Banerjee et al., 2018; Ma et al., 2016). In this study, all keystone taxa belonged to module hubs and connectors (Figure 4; Tables S3 and S4), and major keystones were from phyla *Proteobacteria*, *Actinobacteria*, *Acidobacteria*, *Chloroflexi*, *Ascomycota*, and *Basidiomycota*. These keystones have closed related to other microbial taxa and have a strong influence on the microbial community (Xiao et al., 2022). *Proteobacteria* and *Actinobacteria* are considered to participate in energy metabolisms, such as soil fertility and plant growth (Dai et al., 2018). *Acidobacteria* and *Chloroflexi* are involved in plant residue polymer decomposition (Eichorst et al., 2018). *Bacteroidete* has been reported to play critical roles in C and N metabolism, such as the turnover of carbohydrates, amino acids, and polysaccharides (Han et al., 2017). *Rhodospirillaceae* and *Rhizobiales* have a strong metabolic capacity in the N cycle (Starke et al., 2016). The fungal keystones have different potential roles in the network. The fungal members of *Ascomycota* mediate most of the network modules and are involved in complex substrates decomposition. The highest node degrees were *Dothideomycetes* (class) and *Pezizomycetes* (class), and both belong to *Ascomycota*. This result highlights that these keystones may improve the ecological functions and stability of the karst tiankeng ecosystem.

## 5 CONCLUSIONS

This study investigated the distributing patterns and functional profiles of bacteria and fungi of karst tiankeng with different degrees of degradation and provides insight into the microbial diversity of the karst tiankeng ecosystem. There were significant differences in microbial communities among the three karst tiankeng (nondegraded, moderately degraded, and heavily degraded tiankeng); the bacteria and fungi have differential responses to habitat changes in karst tiankeng. The fungal richness of nondegraded tiankeng is significantly higher than that of degraded tiankeng. The bacterial network was more complex and stable at the nondegraded tiankeng, and tiankeng degradation affects the stability of the bacterial network. The fungal network had more complex and closer relationships at the moderately degraded tiankeng, which may be related to higher plant diversity. In karst tiankeng, the role of keystones in maintaining the soil function and stability is more prominent. The potential function prediction analysis showed that microbial communities in degraded tiankeng may respond to habitat changes by increasing C cycle genes. The ecological value of karst tiankeng is seriously underestimated, and this study contributes to a comprehensive understanding of the ecology of karst tiankeng.

## AUTHOR CONTRIBUTIONS

**Cong Jiang:** Data curation (lead); formal analysis (lead); investigation (lead); writing – original draft (lead). **Hui Zeng:** Conceptualization (lead); writing – review and editing (lead).

## ACKNOWLEDGEMENTS

None.

## FUNDING INFORMATION

This work was supported by the Shenzhen Fundamental Research Program (GXWD20201231165807007‐20200812142216001).

## CONFLICT OF INTEREST

No conflict of interest.

## Supporting information


Appendix S1

## Data Availability

Data from the manuscript is available in the Figshare (https://doi.org/10.6084/m9.figshare.21511473).
