# Supplementary material for: Comparison of soil microbial community structure and function for karst tiankeng with different degrees of degradation
Source: Ecol Evol. 2022 Dec 8;12(12):e9615. doi: 10.1002/ece3.9615 (PMC9731917; doi:10.1002/ece3.9615)
Supplement: Supplementary file 1 — Appendix S1 [file ECE3-12-e9615-s001.docx]

APPENDIX 1


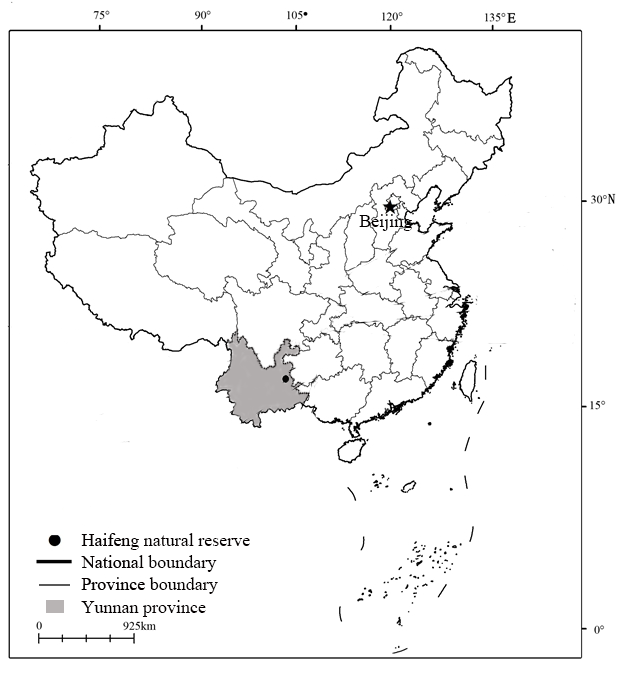


**Fig. S1.** Location of the study site on the map of China.


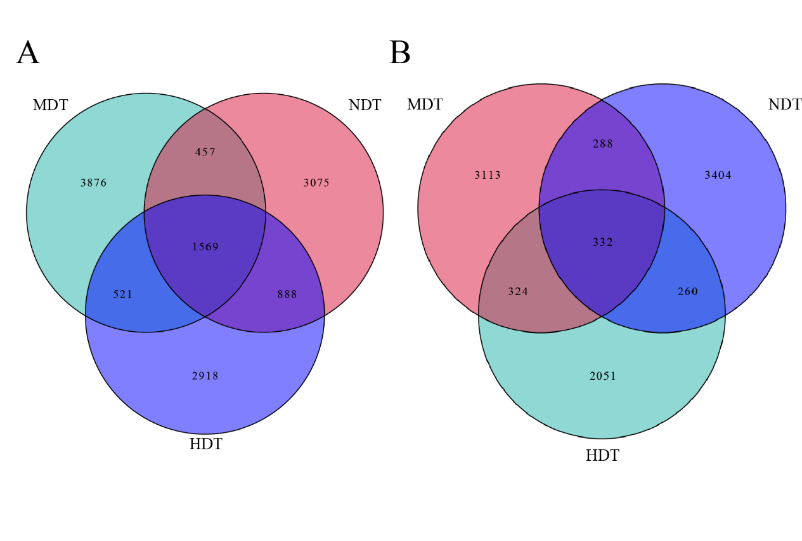


**Fig. S2.** The veen diagram of the unique and shared ASVs of karst tiankeng with different degrees of degradation; (A) bacteria; (B) fungi.


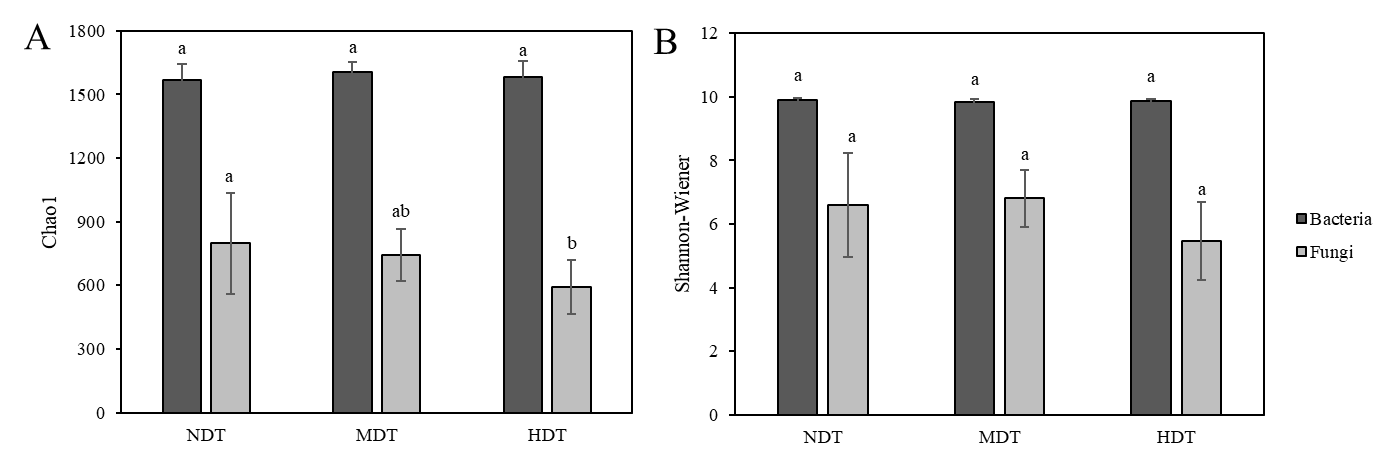


**Fig. S3.** The Chao1 richness (A) and Shannon-Wiener (B) index of bacteria and fungi in karst tiankeng with different degrees of degradation. The error bars represent standard deviation. Different lowercase letters shows statistically significant difference.


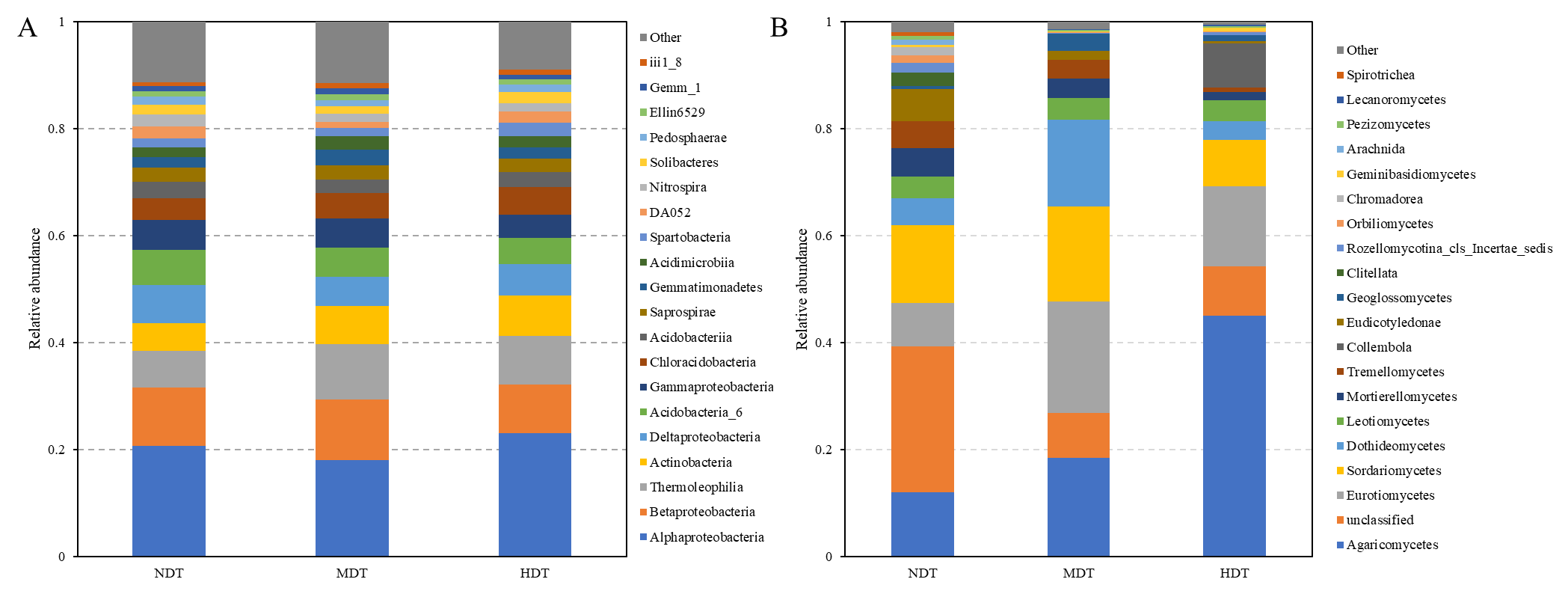


**Fig. S4.** The microbial community composition of major taxa (at the class level) of bacteria (A) and fungi (B) in karst tiankeng with different degrees of degradation.


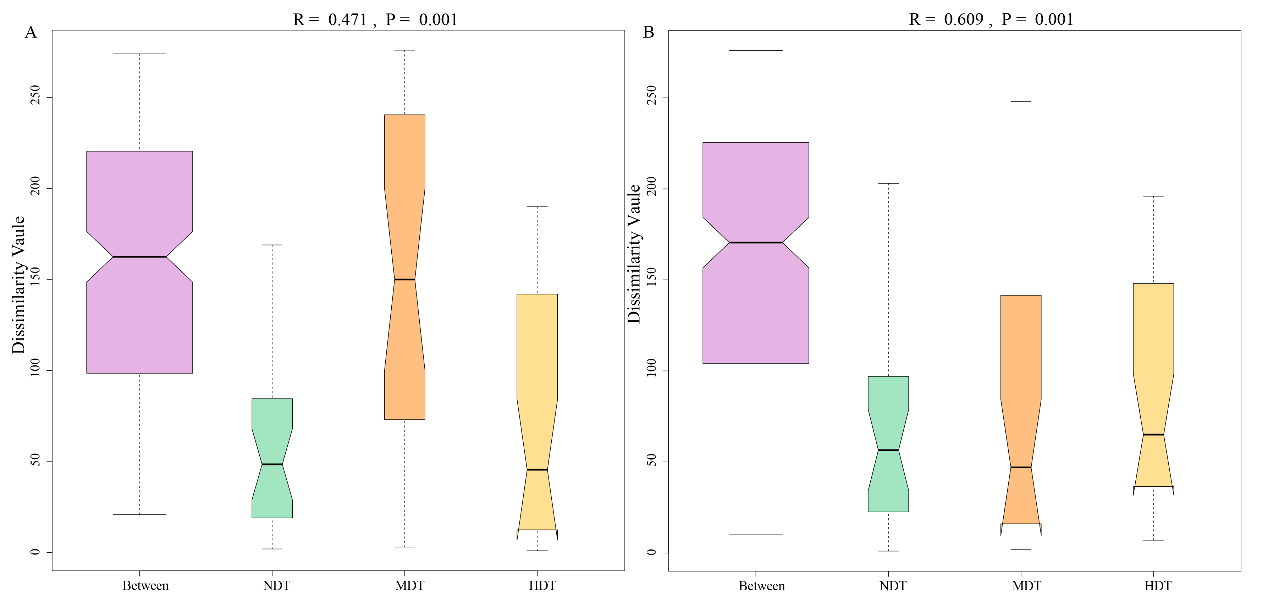


**Fig. S5.** The analysis of similarities (AMOSIM) of bacteria (A) and fungi (B) in karst tiankeng with different degrees of degradation. The AMOSIM analysis was used to test for differences between different groups. P indicates the significance, and an R closer to 1 indicates that the difference among karst tiankeng with different degrees of degradation was significantly higher than the difference within groups.


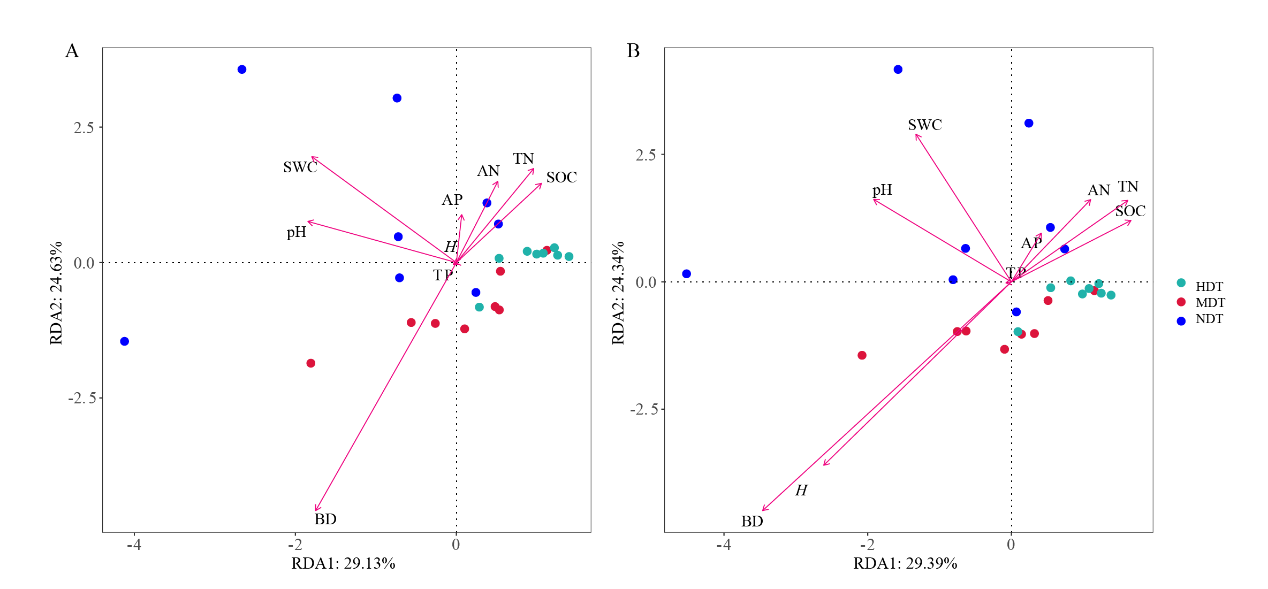


**Fig. S6.** The redundancy analysis (RDA) of bacteria (A) and fungi (B) in karst tiankeng with different degrees of degradation. BD: Soil bulk density; SWC: Soil water content; SOC: Soil organic carbon; TN: Total nitrogen; AN: Available nitrogen; TP: Total phosphorus; AP: Available phosphorus; *H*: Shannon-Wiener index; NDT: Non-degraded tiankeng; MDT: Moderately degraded tiankeng; HDT: Heavily degraded tiankeng.


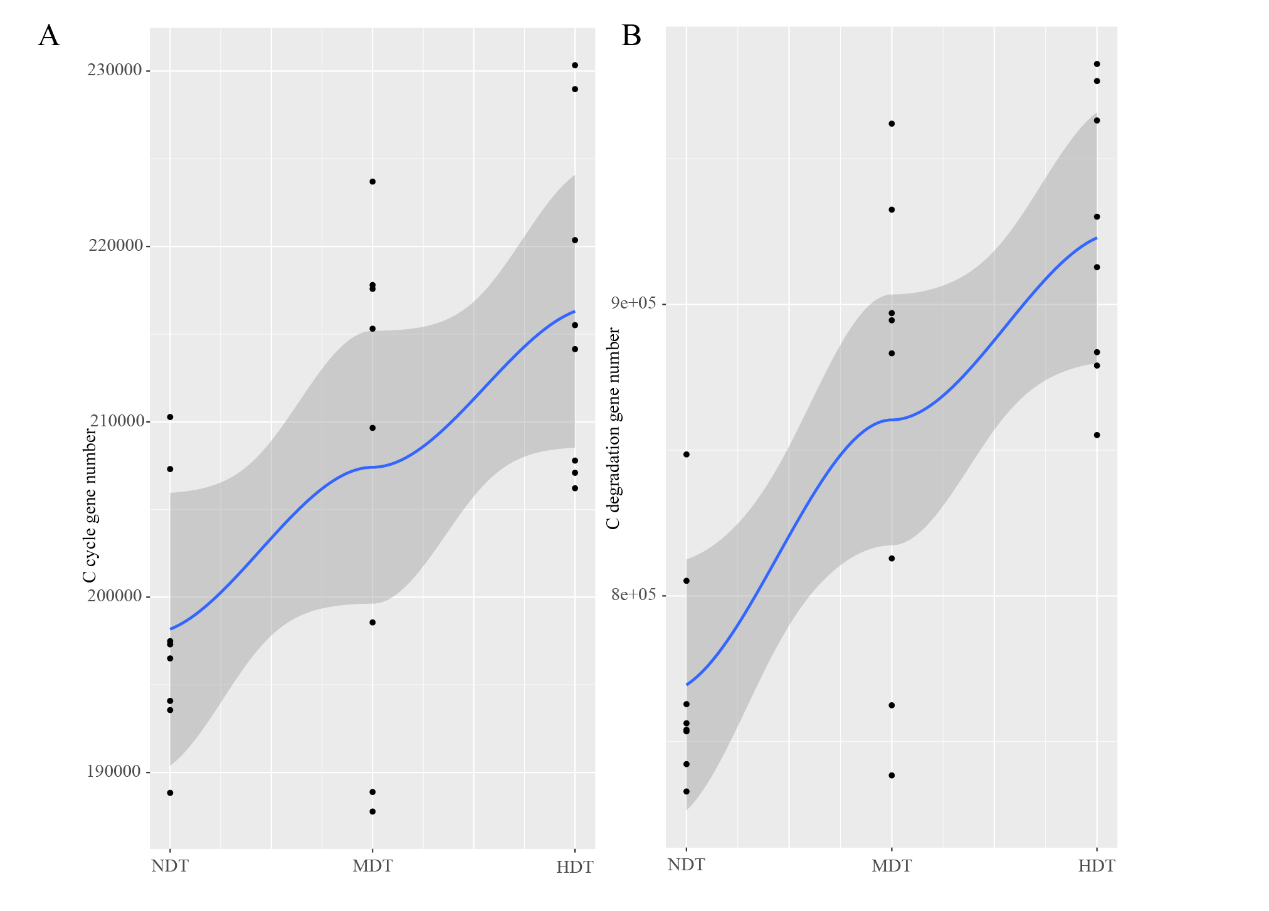


**Fig. S7** The regression analysis of the association between different degradation of karst tiankeng and the number of C cycle gene (A) and C degradation gene (B). C cycle and C degradation genes from the KEGG database. All regressions were significant at the P < 0.01 level.

**Table S1**

Descriptive statistics for vegetation communities feature of karst tiankeng with different degrees of degradation.

|  | *H* | *D* | Main plant species |
| --- | --- | --- | --- |
| NDT | 1.95±0.22b | 1.77±0.29c | *Trachycarpus fortunei* (Hook.) H. Wendl.  *Carpinus monbeigiana* Hand.-Mazz. |
| MDT | 2.31±0.35a | 2.89±0.44a | *Quercus variabilis*  *Alangium chinense*  *Cyclobalanopsis glauca* |
| HDT | 1.84±0.27b | 2.18±0.40b | *Swida oblonga*  *Cyclobalanopsis glauca* |

*H*: Shannon-Wiener index; *D*: Margalef richness index. Different lowercase letters shows statistically significant difference (*P* < 0.05).

**Table S2**

The Mantel test was used to discern correlations among the microbial community (bacterial and fungal), soil physicochemical characteristics, and vegetation characteristics. * indicates *P* < 0.05; ** indicates *P* < 0.01.

|  | Soil characteristics | Bacterial communities | Fungal communities |
| --- | --- | --- | --- |
| Soil characteristics | 1 |  |  |
| Bacterial communities  Fungal communities | 0.2157*  0.4523** | 1  0.1750 | 1 |

**Table S3**

Topological indices of keystone in the bacterial networks.

|  | Generalists | Node information | node.degree | node.betw | node.stress | CC | No. module | Zi | Pi |
| --- | --- | --- | --- | --- | --- | --- | --- | --- | --- |
| NDT | Module hubs | k__Bacteria;p__Chloroflexi;c__TK17;o__mle1_48 | 27 | 24908.87 | 1070038 | 0.06 | 2 | 4.26 | 0.27 |
|  |  | k__Bacteria;p__Chloroflexi;c__Ellin6529 | 15 | 9477.67 | 466879 | 0.10 | 8 | 3.40 | 0 |
|  |  | k__Bacteria;p__Acidobacteria;c__[Chloracidobacteria];o__RB41 | 9 | 3034.32 | 172093 | 0 | 9 | 3.33 | 0.20 |
|  |  | k__Bacteria;p__Proteobacteria;c__Alphaproteobacteria;o__Ellin329 | 51 | 7465.36 | 3486259 | 0.11 | 1 | 3.24 | 0 |
|  |  | k__Bacteria;p__Actinobacteria;c__Actinobacteria;o__Actinomycetales;f__Pseudonocardiaceae;g__Pseudonocardia | 20 | 12253.11 | 759305 | 0.11 | 2 | 3.11 | 0.19 |
|  |  | k__Bacteria;p__Acidobacteria;c__DA052;o__Ellin6513 | 47 | 3938.75 | 2564336 | 0.09 | 1 | 2.92 | 0 |
|  |  | k__Bacteria;p__Proteobacteria | 46 | 4074.50 | 2572715 | 0.09 | 1 | 2.84 | 0 |
|  |  | k__Bacteria;p__Proteobacteria;c__Betaproteobacteria | 46 | 5450.98 | 2649074 | 0.09 | 1 | 2.84 | 0 |
|  |  | k__Bacteria;p__Acidobacteria;c__DA052;o__Ellin6513 | 46 | 10115.24 | 3054075 | 0.06 | 1 | 2.76 | 0.04 |
|  |  | k__Bacteria;p__Acidobacteria;c__Acidobacteriia;o__Acidobacteriales;f__Acidobacteriaceae | 45 | 10992.87 | 3890203 | 0.07 | 1 | 2.76 | 0 |
|  |  | k__Bacteria;p__WS3;c__PRR_12;o__Sediment_1 | 10 | 5493.36 | 899346 | 0 | 3 | 2.71 | 0 |
|  |  | k__Bacteria;p__Bacteroidetes;c__[Saprospirae];o__[Saprospirales];f__Chitinophagaceae | 10 | 5493.38 | 899346 | 0 | 3 | 2.71 | 0 |
|  |  | k__Bacteria;p__Acidobacteria;c__[Chloracidobacteria];o__RB41 | 44 | 3208.64 | 2491786 | 0.06 | 1 | 2.67 | 0 |
|  |  | k__Bacteria;p__Proteobacteria;c__Alphaproteobacteria;o__Rhizobiales;f__Hyphomicrobiaceae;g__Pedomicrobium | 43 | 10116.66 | 3390731 | 0.02 | 1 | 2.59 | 0 |
|  |  | k__Bacteria;p__Proteobacteria;c__Betaproteobacteria;o__MND1 | 45 | 17761.97 | 3247208 | 0.06 | 1 | 2.59 | 0.09 |
|  |  | k__Bacteria;p__Actinobacteria;c__Thermoleophilia;o__Gaiellales;f__Gaiellaceae | 7 | 2169.39 | 395383 | 0.38 | 10 | 2.58 | 0 |
|  | Connectors | k__Bacteria;p__Acidobacteria;c__[Chloracidobacteria];o__RB41 | 8 | 16842.93 | 847622 | 0.04 | 2 | -0.12 | 0.66 |
|  |  | k__Bacteria;p__Acidobacteria;c__Solibacteres;o__Solibacterales;f__Solibacteraceae;g__Candidatus_Solibacter | 6 | 8778.32 | 1138024 | 0 | 1 | -0.72 | 0.67 |
|  |  | k__Bacteria;p__Proteobacteria;c__Alphaproteobacteria;o__Rhodospirillales;f__Rhodospirillaceae | 3 | 3952.48 | 201306 | 0 | 1 | -0.80 | 0.67 |
|  |  | k__Bacteria;p__Actinobacteria;c__Acidimicrobiia;o__Acidimicrobiales | 3 | 2513.29 | 148162 | 0 | 2 | -0.81 | 0.67 |
|  |  | k__Bacteria;p__Proteobacteria;c__Betaproteobacteria;o__Burkholderiales;f__Comamonadaceae | 4 | 11919.84 | 612951 | 0 | 6 | -0.89 | 0.63 |
|  |  | k__Bacteria;p__Proteobacteria;c__Alphaproteobacteria;o__Rhodobacterales;f__Hyphomonadaceae | 4 | 9658.15 | 16729536 | 0 | 7 | -1.21 | 0.63 |
| MDT | Module hubs | k__Bacteria;p__Proteobacteria;c__Betaproteobacteria;o__Burkholderiales;f__Comamonadaceae;g__Hydrogenophaga | 9 | 3351.25 | 270829 | 0.22 | 10 | 2.74 | 0 |
|  |  | k__Bacteria;p__Proteobacteria;c__Alphaproteobacteria;o__Sphingomonadales;f__Sphingomonadaceae;g__Sphingomonas | 17 | 10005.55 | 414078 | 0.35 | 1 | 2.85 | 0.11 |
|  |  | k__Bacteria;p__Actinobacteria;c__Actinobacteria;o__Actinomycetales | 20 | 33752.15 | 7750243 | 0.1 | 8 | 3.78 | 0.19 |
|  |  | k__Bacteria;p__Proteobacteria;c__Alphaproteobacteria;o__Rhizobiales | 14 | 23713.69 | 6015163 | 0.02 | 6 | 2.91 | 0.46 |
|  |  | k__Bacteria;p__Proteobacteria;c__Alphaproteobacteria;o__Rhizobiales;f__Phyllobacteriaceae;g__Mesorhizobium | 15 | 11599.34 | 793120 | 0.13 | 7 | 3.70 | 0.12 |
|  |  | k__Bacteria;p__Proteobacteria;c__Alphaproteobacteria;o__Rhodobacterales;f__Hyphomonadaceae | 10 | 8704.45 | 451298 | 0.13 | 6 | 2.91 | 0 |
|  |  | k__Bacteria;p__Actinobacteria;c__Thermoleophilia;o__Gaiellales;f__Gaiellaceae | 15 | 10873.72 | 663637 | 0.13 | 7 | 3.70 | 0.12 |
|  |  | k__Bacteria;p__Actinobacteria;c__Thermoleophilia;o__Solirubrobacterales;f__Conexibacteraceae | 29 | 48016.68 | 5200088 | 0 | 2 | 3.78 | 0.25 |
|  |  | k__Bacteria;p__Bacteroidetes;c__[Saprospirae];o__[Saprospirales];f__Chitinophagaceae | 19 | 33062.15 | 7734869 | 0.11 | 8 | 3.52 | 0.19 |
|  |  | k__Bacteria;p__Acidobacteria;c__[Chloracidobacteria];o__RB41 | 30 | 44527.28 | 5184910 | 0.02 | 2 | 3.98 | 0.24 |
|  |  | k__Bacteria;p__Proteobacteria;c__Alphaproteobacteria;o__Rhizobiales;f__Hyphomicrobiaceae;g__Rhodoplanes | 8 | 5479.93 | 622909 | 0.04 | 5 | 2.54 | 0 |
| HDT | Module hubs | k__Bacteria;p__Acidobacteria;c__Acidobacteriia;o__Acidobacteriales;f__Koribacteraceae | 23 | 47901.09 | 5043277 | 0.02 | 3 | 6.47 | 0.31 |
|  |  | k__Bacteria;p__Proteobacteria;c__Alphaproteobacteria;o__BD7_3 | 18 | 43076.02 | 2862260 | 0.01 | 2 | 4.69 | 0.60 |
|  |  | k__Bacteria;p__Proteobacteria;c__Betaproteobacteria;o__Burkholderiales;f__Burkholderiaceae;g__Burkholderia;s__glathei | 15 | 29684.13 | 1080354 | 0.01 | 4 | 4.40 | 0.24 |
|  |  | k__Bacteria;p__Proteobacteria;c__Alphaproteobacteria;o__Rhodospirillales;f__Rhodospirillaceae | 10 | 3474.99 | 153256 | 0.04 | 0 | 3.76 | 0 |
|  |  | k__Bacteria;p__Proteobacteria;c__Alphaproteobacteria;o__Caulobacterales;f__Caulobacteraceae;g__Phenylobacterium | 28 | 78355.01 | 5032095 | 0.01 | 5 | 3.71 | 0.56 |
|  |  | k__Bacteria;p__Acidobacteria;c__DA052;o__Ellin6513 | 11 | 11513.13 | 129609 | 0.02 | 9 | 3.32 | 0.31 |
|  |  | k__Bacteria;p__Acidobacteria;c__RB25 | 10 | 4842.02 | 84491 | 0.27 | 10 | 3.26 | 0 |
|  |  | k__Bacteria;p__Proteobacteria;c__Betaproteobacteria;o__Ellin6067 | 12 | 27205.08 | 3888894 | 0.17 | 11 | 3.06 | 0 |
|  |  | k__Bacteria;p__Proteobacteria;c__Deltaproteobacteria;o__Syntrophobacterales;f__Syntrophobacteraceae | 6 | 3473.50 | 1887494 | 0.07 | 12 | 2.85 | 0 |
|  |  | k__Bacteria;p__Actinobacteria;c__Actinobacteria;o__Actinomycetales;f__Micromonosporaceae;g__Pilimelia | 9 | 6405.79 | 394489 | 0.17 | 13 | 2.50 | 0 |
|  | Connectors | k__Bacteria;p__Acidobacteria;c__Acidobacteria_6;o__CCU21 | 3 | 12185.07 | 453354 | 0 | 3 | -0.54 | 0.67 |

**Table S4**

Topological indices of keystone in the fungal networks.

|  | Generalists | Node information | node.degree | node.betw | node.stress | CC | No. module | Zi | Pi |
| --- | --- | --- | --- | --- | --- | --- | --- | --- | --- |
| NDT | Module hubs | k__Fungi;p__Basidiomycota;c__Agaricomycetes;o__Russulales;f__Russulaceae;g__Russula;s__Russula_virescens | 13 | 1230.32 | 6262 | 0.10 | 4 | 3.97 | 0 |
|  |  | k__Fungi;p__Ascomycota;c__Pezizomycetes;o__Pezizales;f__Pyronemataceae;g__Tricharina;s__Tricharina_sp | 36 | 4046.94 | 43838 | 0.07 | 0 | 3.39 | 0.38 |
|  |  | k__Fungi;p__Ascomycota;c__Dothideomycetes;o__Tubeufiales;f__Tubeufiaceae;g__Titaea;s__Titaea_maxilliformis | 39 | 4943.54 | 60120 | 0.08 | 0 | 3.05 | 0.52 |
|  |  | k__Fungi;p__Ascomycota;c__Leotiomycetes;o__Helotiales;f__Myxotrichaceae;g__Oidiodendron;s__Oidiodendron_chlamydosporicum | 12 | 570.94 | 6122 | 0.18 | 4 | 2.67 | 0.28 |
|  | Connectors | k__Fungi;p__Ascomycota;c__Sordariomycetes;o__Hypocreales;f__Nectriaceae;g__Mariannaea | 11 | 3226.75 | 26703 | 0.04 | 3 | 1.39 | 0.68 |
|  |  | k__Fungi;p__Basidiomycota | 3 | 327.08 | 2390 | 0 | 4 | -1.23 | 0.67 |
|  |  | k__Fungi;p__Ascomycota;c__Eurotiomycetes;o__Onygenales | 3 | 1132.07 | 10300 | 0 | 3 | -1.26 | 0.67 |
|  |  | k__Fungi;p__Basidiomycota | 5 | 543.89 | 9678 | 0 | 1 | -0.40 | 0.64 |
|  |  | k__Fungi;p__Basidiomycota;c__Agaricomycetes;o__Agaricales;f__Clavariaceae;g__Clavulinopsis;s__Clavulinopsis_sp | 4 | 272.63 | 5226 | 0 | 1 | -0.40 | 0.63 |
| MDT | Module hubs | k__Fungi;p__Ascomycota;c__Sordariomycetes;o__Hypocreales;f__Nectriaceae;g__Thelonectria;s__Thelonectria_sp | 31 | 2114.14 | 24202 | 0.13 | 1 | 4.09 | 0.12 |
|  |  | k__Viridiplantae;p__Anthophyta;c__Eudicotyledonae;o__Lamiales;f__Acanthaceae;g__Justicia | 18 | 2020.72 | 40105 | 0.13 | 0 | 3.07 | 0 |
|  |  | k__Fungi | 32 | 809.03 | 7904 | 0.12 | 1 | 2.75 | 0.49 |
|  | Connectors | k__Fungi;p__Ascomycota;c__Dothideomycetes;o__Pleosporales | 13 | 165.17 | 1922 | 0.22 | 6 | -0.63 | 0.71 |
|  |  | k__Fungi;p__Ascomycota;c__Dothideomycetes;o__Pleosporales;f__Sporormiaceae;g__Sporormiella;s__Sporormiella_minima | 16 | 276.70 | 6426 | 0.25 | 2 | -0.04 | 0.70 |
|  |  | k__Fungi;p__Ascomycota;c__Dothideomycetes;o__Pleosporales;f__Sporormiaceae;g__Preussia;s__Preussia_lignicola | 16 | 503.08 | 6416 | 0.17 | 5 | -0.15 | 0.70 |
|  |  | k__Fungi;p__Ascomycota;c__Dothideomycetes;o__Capnodiales;f__Cladosporiaceae;g__Cladosporium | 28 | 1586.48 | 21724 | 0.17 | 2 | 1.57 | 0.67 |
|  |  | k__Fungi;p__Ascomycota;c__Eurotiomycetes;o__Chaetothyriales;f__Trichomeriaceae;g__Knufia;s__Knufia_sp | 3 | 90.35 | 886 | 0 | 2 | -1.20 | 0.67 |
|  |  | k__Fungi;p__Ascomycota | 9 | 109.34 | 1269 | 0.44 | 1 | -0.69 | 0.64 |
|  |  | k__Fungi;p__Ascomycota;c__Sordariomycetes;o__Hypocreales;f__Nectriaceae;g__Fusarium;s__Fusarium_redolens | 29 | 2283.1 | 25571 | 0.17 | 2 | 2.04 | 0.63 |
|  |  | k__Fungi;p__Ascomycota;c__Eurotiomycetes;o__Chaetothyriales | 8 | 116.14 | 2148 | 0.32 | 5 | -0.94 | 0.63 |
|  |  | k__Fungi;p__Ascomycota;c__Sordariomycetes;o__Chaetosphaeriales;f__Chaetosphaeriaceae;g__Chloridium | 4 | 459.62 | 3812 | 0 | 2 | -0.96 | 0.63 |
| HDT | Module hubs | k__Fungi;p__Ascomycota;c__Eurotiomycetes;o__Chaetothyriales;f__Trichomeriaceae;g__Knufia;s__Knufia_sp | 11 | 2212.61 | 17719 | 0.18 | 0 | 3.01 | 0 |
|  |  | k__Fungi;p__Basidiomycota;c__Agaricomycetes;o__Sebacinales;f__Sebacinaceae;g__Sebacina;s__Sebacina_sp | 7 | 1652.81 | 4827 | 0.05 | 8 | 2.60 | 0.45 |
|  |  | k__Fungi;p__Basidiomycota;c__Agaricomycetes;o__Agaricales;f__Agaricaceae;g__Agaricus;s__Agaricus_xanthodermus | 8 | 931.30 | 4915 | 0.25 | 2 | 2.59 | 0 |
|  |  | k__Fungi;p__Basidiomycota | 8 | 931.30 | 4915 | 0.25 | 2 | 2.59 | 0 |
|  |  | k__Fungi;p__Ascomycota;c__Dothideomycetes;o__Dothideomycetes_ord_Incertae_sedis;f__Eremomycetaceae;g__Arthrographis;s__Arthrographis_arxii | 14 | 4322.02 | 38050 | 0.11 | 1 | 2.51 | 0.52 |
|  | Connectors | k__Fungi;p__Basidiomycota;c__Agaricomycetes;o__Agaricales;f__Agaricaceae;g__Agaricus;s__Agaricus_xanthodermus | 10 | 5310.61 | 43943 | 0.04 | 4 | 0.92 | 0.74 |
|  |  | k__Fungi;p__Rozellomycota;c__Rozellomycotina_cls_Incertae_sedis;o__GS11 | 8 | 2490.63 | 20710 | 0.04 | 6 | 0.10 | 0.63 |
|  |  | k__Fungi;p__Basidiomycota;c__Agaricomycetes;o__Russulales;f__Russulaceae;g__Russula;s__Russula_cyanoxantha | 4 | 767.05 | 5527 | 0 | 6 | -0.69 | 0.63 |

**Table S5**

The predicted KEGG categories abundance of karst tiankeng with different degrees of degradation.

|  | NDT | MDT | HDT |
| --- | --- | --- | --- |
| Cell growth and death | 2.066±0.019a | 1.977±0.025b | 2.051±0.015a |
| Cell motility | 3.051±0.070a | 3.013±0.093a | 2.973±0.074a |
| Cellular community - prokaryotes | 1.472±0.014a | 1.492±0.050a | 1.436±0.010b |
| Membrane transport | 1.311±0.013ab | 1.316±0.038a | 1.287±0.011b |
| Signal transduction | 1.006±0.005a | 1.014±0.011a | 0.997±0.004b |
| Folding, sorting and degradation | 2.599±0.030a | 2.524±0.044b | 2.520±0.027b |
| Replication and repair | 4.000±0.021a | 3.947±0.042b | 3.926±0.022b |
| Translation | 2.346±0.034a | 2.271±0.049b | 2.260±0.033b |
| Drug resistance: antimicrobial | 1.460±0.008a | 1.437±0.012b | 1.431±0.006b |
| Amino acid metabolism | 11.257±0.044c | 11.434±0.068a | 11.340±0.048b |
| Biosynthesis of other secondary metabolites | 6.042±0.044a | 5.978±0.074a | 6.018±0.057a |
| Carbohydrate metabolism | 9.185±0.043c | 9.306±0.040a | 9.253±0.042b |
| Chemical structure transformation maps | 2.331±0.061b | 2.290±0.073b | 2.422±0.067a |
| Energy metabolism | 4.034±0.016a | 3.974±0.026b | 3.968±0.016b |
| Global and overview maps | 5.695±0.027a | 5.682±0.031a | 5.677±0.026a |
| Glycan biosynthesis and metabolism | 2.938±0.033a | 2.793±0.089b | 2.876±0.063a |
| Lipid metabolism | 6.222±0.038b | 6.377±0.072a | 6.366±0.035a |
| Metabolism of cofactors and vitamins | 9.395±0.058a | 9.185±0.096b | 9.225±0.058a |
| Metabolism of other amino acids | 6.677±0.023a | 6.647±0.023b | 6.657±0.021ab |
| Metabolism of terpenoids and polyketides | 3.637±0.054c | 3.868±0.108a | 3.778±0.041b |
| Nucleotide metabolism | 1.174±0.011a | 1.152±0.015b | 1.148±0.011b |
| Xenobiotics biodegradation and metabolism | 5.575±0.107b | 5.823±0.168a | 5.848±0.110a |
| Endocrine system | 0.977±0.008b | 1.004±0.012a | 1.010±0.006a |

Different lowercase letters shows statistically significant difference (*P* < 0.05).
